# RAGE Signaling in Melanoma Tumors

**DOI:** 10.3390/ijms21238989

**Published:** 2020-11-26

**Authors:** Olamide T. Olaoba, Sultan Kadasah, Stefan W. Vetter, Estelle Leclerc

**Affiliations:** Department of Pharmaceutical Sciences, School of Pharmacy, North Dakota State University, Fargo, ND 58105, USA; olaobamide@gmail.com (O.T.O.); sultan.kadasah@ndsu.edu (S.K.); Stefan.Vetter@ndsu.edu (S.W.V.)

**Keywords:** melanoma, RAGE, receptor for advanced glycation end products, S100 proteins, HMGB1, inflammation, tumorigenesis, melanomagenesis

## Abstract

Despite recent progresses in its treatment, malignant cutaneous melanoma remains a cancer with very poor prognosis. Emerging evidences suggest that the receptor for advance glycation end products (RAGE) plays a key role in melanoma progression through its activation in both cancer and stromal cells. In tumors, RAGE activation is fueled by numerous ligands, S100B and HMGB1 being the most notable, but the role of many other ligands is not well understood and should not be underappreciated. Here, we provide a review of the current role of RAGE in melanoma and conclude that targeting RAGE in melanoma could be an approach to improve the outcomes of melanoma patients.

## 1. Melanoma

Melanoma originates from the abnormal growth of melanocytes, and it can become very invasive and aggressive [1]. Despite being relatively rare among cutaneous cancers (<5%), melanoma is the leading cause of skin cancer-related mortality [2,3]. Melanocytes are part of a complex of three cell types that constitute the keratinocyte, Langerhans cells, and melanocyte (KLM) unit of the epidermis, and they are critical for melanin production [4]. Melanocytes synthesize melanin within special organelles called melanosomes. Melanin production is a process that is regulated by UV radiation, synthesis of the melanocyte stimulating hormone (MSH) and increased expression of its receptor, the melanocortin 1 receptor (MC1-R). A key enzyme in the synthesis of melanin pigments is tyrosinase [5]. Upon melanin synthesis, the melanosomes are transferred to keratinocytes through the help of dendrites [4]. In the skin, melanin has a protective role and provides a photo shielding effect against DNA-damaging UV radiation. Additionally, melanin has chemoprevention, thermoregulation, and metal-chelating properties [5]. In the skin and other tissues, the loss of melanocytes is associated with pathological consequences [4,6].

### 1.1. Driver Mutations in Melanomagenesis

Melanomagenesis is the result of genetic and epigenetic modifications, as well as alterations in signaling pathways controlling key cellular functions. Next-generation sequencing analysis of 686 cutaneous melanoma tissues revealed key genes in melanomagenesis [7]. Three important pathways contributing to melanomagenesis were found to be the cyclin-dependent kinase inhibitor 2A (CDKN2A) pathway, the mitogen-activated protein kinase (MAPK) pathway including neuroblastoma RAS viral oncogene homolog (NRAS) and V-raf murine sarcoma viral oncogene homolog B1 (BRAF), as well as the phosphatidylinositol-3 kinase (PI3K)/AKT/mammalian target of rapamycin (mTOR) pathway [7,8,9]. In this large study, the most frequent alterations were found in BRAF (49.6%) and RAS (29.4%), with 94% RAS mutations being in NRAS. In addition to these two main melanoma driver genes, other driver genes were identified and classified into three groups based on their mutation prevalence. The first group contained genes that were found mutated in 10 to 20% of melanoma tissues and included CDKN2A, neurofibromatosis type1 (NF1), AT-rich interactive domain-containing protein 2 (ARID2), and tumor protein p53 (TP53). The second group comprised of serine/threonine-protein phosphatase 6 catalytic subunit (PPP6C), DEAD-box helicase 3 X-linked (DDX3X), phosphatase and tension homology (PTEN), and ras-related C3 botulinum toxin substrate 1 (RAC1) genes, which showed mutations in 5 to 9% melanoma tissues. F-box/WD repeat-containing protein 7 (FBXW7), sorting nexin 31 (SNX31), phosphatidylinositol-3,4,5- trisphosphate-dependent rac exchange factor 2 (PREX2), MAPK1-2, transforming acidic coiled-coil-containing protein 1 (TACC1), KIT tyrosine protein kinase (KIT), isocitrate dehydrogenase1. (IDH1), retinoblastoma protein 1 (RB1), splicing factor 3b subunit 1 (SF3B1), catenin (cadherin-associate protein) beta 1 (CTNMB1), PIK3 catalytic subunit alpha (PI3KCA), cyclin-dependent kinase 4 (CDK4), ras p21 protein activator 2 (RASA2), Wilms’ tumor suppressor gene 1 (WT1), enhancer of zeste homolog 2 (EZH2), and serine/threonine-protein kinase 19 (STK19) genes constituted the third group, and mutations in these genes were less frequently detected (<5%). It was also observed that 6.6% of cutaneous melanoma tissues were negative for any genetic modification [7]. This study supported the current concept that the mutational landscape of genes in melanomas is unparalleled, and thus, it results in large molecular heterogeneity.

### 1.2. Cutaneous and Non-Cutaneous Melanoma

Although most melanoma tumors develop in the skin (cutaneous melanoma), they can also arise in mucosal membranes, [10,11,12,13,14,15,16,17], in the eye (uveal melanoma) [18], and primary melanoma tumors have also been described in leptomeninges (Table 1) [19]. Among these different types of melanoma, cutaneous melanoma is by far the most prevalent. For instance, the histopathological diagnosis of malignant melanoma in India during a period of 5 years showed that 93.4% of the cases were cutaneous melanoma, whereas only 6.6% non-cutaneous melanoma were characterized in conjunctiva, anorectum, gingiva-buccal sulcus, vagina, palate, and the nasal cavity [20]. Although cutaneous melanoma has a higher likelihood in Caucasians than in other ethnicities, mucosal melanoma incidence is not race dependent [21]. Features such as the etiology, pathogenesis, epidemiology, prognosis, clinical course, and frequency of genetic alterations further distinguish mucosal melanoma from its cutaneous counterpart [22,23]. Mucosal melanoma is frequently present in the head and neck region (55.4% all cases), vulvovaginal area (18%), and anal/rectal region (23.8%), and it can be found in the urinary tract as well (2.8%) [23,24,25]. In ocular melanoma, the most common sites for melanoma are the posterior uvea, affecting the ciliary body and choroid [26]. However, melanoma in the conjunctiva, orbit, retina, vitreous, iris, and the anterior chamber of the eye have also been reported [27]. The other non-cutaneous melanoma, such as the poorly prognosed leptomeningeal melanoma may emerge from severe neurocutaneous melanocytosis [19]. Cutaneous melanoma can be classified in four major groups according to histopathological features: superficial spreading melanoma (SSM), nodular, lentigo, and acral lentigious melanomas [28]. SSM is the most common group and accounts for 70% of all cutaneous melanoma cases.

### 1.3. Staging of Melanoma and Patient Survival

The staging system of melanoma established by the American Joint Commission in Cancer (AJCC) was recently revised and updated [29]. This system is based on four pathological stage groupings (I to IV) and different factors such as the primary tumor thickness and volume, whether the tumor is ulcerated or has reached the nearby lymph nodes, the presence of distant metastases, or the mitotic index of tumors [29,30].

Among the four types of melanoma (cutaneous, mucosal, ocular, and leptomeningeal), staging is less difficult with cutaneous melanoma than non-cutaneous ones because of the presence of tumors on the skin. For similar reasons, patient survival is higher in this type of melanoma than in the non-cutaneous counterparts because of earlier diagnosis. For instance, a recent meta-analysis showed a 2.25-fold higher lethality in mucosal than in cutaneous melanoma [31]. The overall survival (OS) and disease-specific survival (DSS) in melanoma patients depends on many factors including tumor stage, histology, type of treatment the patient is receiving, as well as age [32]. Overall, metastatic melanoma is very aggressive and poorly controlled; it is associated with low OS, usually between 6 and 9 months [33].

### 1.4. Melanoma Biomarkers

A biomarker (molecule) is a molecule that can be measured in tissues, blood, and other body fluids and is an indicator of a disease [34]. Biomarkers can be used for diagnostic or prognosis purposes. Diagnostic biomarkers are present in higher levels in diseased patients than healthy patients. Prognostic or predictive markers have increased expression in advanced stages of the disease or different expression during treatment and can indicate potential recurrence of the disease [34]. Although several molecules have some potential clinical values as melanoma biomarkers (lactate dehydrogenase (LDH), tyrosinase, Programmed Cell Death 1 Ligand 1 (PD1L1) and S100B), they also presented some limitations, and for this reason, there is currently no ideal biomarker in melanoma [35,36,37,38]. Here, we will just briefly discuss the strengths and limitations of these four molecules with potential clinical values. LDH is probably the strongest independent prognostic and main serum biomarker for clinical use in metastatic cancer patients [39,40,41]. LDH catalyzes the conversion of pyruvate into lactate, which is a reaction that occurs when oxidative phosphorylation is impaired, such as observed in cancer tumors through a process described as the Warburg effect [42]. This process is further enhanced in hypoxic regions of solid tumors due to poor vascularization and supplied oxygen, and it is observed in melanoma tumors [43,44]. In metastatic melanoma patients, elevated levels of serum LDH, measured using its enzymatic activity, correlate with low overall survival [45] and also appear to be strongly predictive of overall survival following immunotherapy with the current standards of care ipilimubab, pembrolizumab, and nivolumab [46,47]. However, elevated levels of LDH are not specific to melanoma tumors and are also observed in other diseased tissues [48].

Tyrosinase is another molecule with clinical value in melanoma. Tyrosinase is an enzyme that participates in the synthesis of melanin pigments in melanocytes and melanoma. In melanoma patients, serum levels of circulating tyrosinase mRNA transcripts have been evaluated as a prognostic marker: high expression levels are associated with poor prognosis [35,49,50]. However, a large variability has been observed between studies due in part to the transient presence of melanoma tumor cells in the blood stream and to non-standardized protocols when performing polymerase chain (PCR) reaction experiments [51].

Another molecule with potential clinical value is PD1L1, the ligand activating Programmed Cell Death Receptor 1 (PD-1) [52]. PD1L1 is a cell surface transmembrane protein expressed by tumor cells and can also occur in a soluble form as result of alternate splicing or proteolysis. PD-1 is a cell surface receptor expressed by immune cells (T and B cells, macrophages) that transmits apoptotic or activation signals, resulting in either the suppression or activation of immune cells. In cytotoxic T cells, the PD-1/PD1L1 axis acts as a switch that turns off cytotoxic T-cell activation, resulting in tumor cells that are capable of evading immune surveillance [53]. High levels of PD1L1 are found in certain types of cancer, including melanoma [53,54]. Expression levels of PD1L1 in tumor biopsies are thought to predict the response levels of melanoma patients to treatment with immune checkpoint inhibitors, such as nivolumab and pembrolizumab (PD-1 antibodies) [55,56]. However, there are some limitations regarding the use of PD1L1 levels as biomarkers for immunotherapy. These limitations reside in the heterogeneity of PD1L1 expression in melanoma tumors [57] and the observation that even patients carrying melanoma tumors with a low expression of PD1L1 have shown benefits from immunotherapy [58,59].

S100B has clinical value as a prognostic biomarker of treatment response. S100B is a small EF-hand calcium binding protein that is expressed by melanocytes and released in the extracellular milieu by melanoma tumors [60]. Inside cells, S100B interacts with the transcription factor p53 and inhibits its transcriptional activity, resulting in the increased survival of melanoma cells by decreasing p53-dependent apoptosis [61]. When secreted by tumors, S100B can be used as a prognostic biomarker, higher levels of S100B being predictive of poorer outcome [62,63,64]. S100B also appears to be a promising biomarker for treatment response and overall survival in melanoma patients treated with immune checkpoints inhibitors (anti PD-1 antibodies) [65].

### 1.5. Treatment of Cutaneous Melanoma

Tumor heterogeneity [66] makes the treatment of melanoma tumors very challenging [67,68]. In the early stages of the disease, the most effective treatment is surgical resection of the primary tumor, and in these patients, 5-year survival exceeds 95%. However, once it forms metastases, melanoma becomes a very aggressive cancer and without treatment, patients survive less than one year [69].

For many years, patients with metastatic melanoma had very few treatment options. One option was the cytotoxic agent dacarbazine, and two other options were the immunotherapeutic agents interleukin 2 (IL-2) and interferon α (IFN-α). Treatment with either dacarbazine or IL-2 resulted in low response rates (<20%) and transient effects, and it was associated with severe adverse effects. In addition, none of these agents was shown to significantly prolong the overall survival of patients [69]. Some encouraging results with increased overall survivals were observed with interferon a (IFN-α), but the effects were sub-optimal, and the adverse events were severe for most patients [69]. Significant improvements in metastatic melanoma therapy occurred in the last decade with the approval of new drugs for targeted therapy (BRAF and MEK inhibitors) and immunotherapy (immune check-points inhibitors) (Table 2) [70].

Vemurafenib, dabrafenib, and encorafenib inhibit the mutant forms of the BRAF kinase, where valine in position 600 is replaced by an aspartic acid (V600E) or a lysine residue (V600K). These mutations are frequently observed in melanoma tumors, with BRAF V600E mutants being present in up to 60% of melanoma tumors [71]. Trametinib and binimetinib are MEK inhibitors and used in combination with BRAF mutant inhibitors [72,73,74,75]. Although the overall survival of melanoma patients has improved with kinase inhibitors, many patients experienced recurrence of the disease due to different mechanisms of resistance [76]. Three antibodies—ipilimumab, nivolumab, and pembrolizumab—have been approved by the FDA for the treatment of metastatic melanoma (Table 2). These antibodies target two major immunosuppressive checkpoints: the cytotoxic T lymphocyte antigen 4 protein (CTLA-4) (ipilimumab) and PD-1 (nivolumab and pembrolizumab) [77]. Among these antibodies, nivolumab and pembrolizumab present higher efficacy and safety than ipilimumab [77]. Although these new immunotherapeutic agents result in improved outcomes for patients, compared to other melanoma therapeutic agents, they are also associated with severe adverse effects and are only suitable for the more fit patients [77].

Despite the important progresses made toward the treatment of metastatic melanoma, the treatment outcomes are still not satisfactory, and it is urgent to continue to improve existing treatments or to develop new therapeutic strategies. As we will discuss in this review, an increasing number of experimental evidences suggests that the receptor for advance glycation end products (RAGE) could be a relevant therapeutic target for the treatment of metastatic melanoma. The next sections of this review will provide information on the role of RAGE in melanoma.

## 2. RAGE

### 2.1. RAGE Structure and Isoforms

RAGE is a single transmembrane domain multi-ligand cell surface receptor belonging to the immunoglobulin (Ig) superfamily and is encoded in the class III Major Histocompatibility Complex (MHC) at position 6p21.3 [86,87,88,89]. This chromosomal region contains multiple genes involved in inflammatory and immune disorders, suggesting that RAGE plays a role in inflammation as well [90].

**Figure 1:**
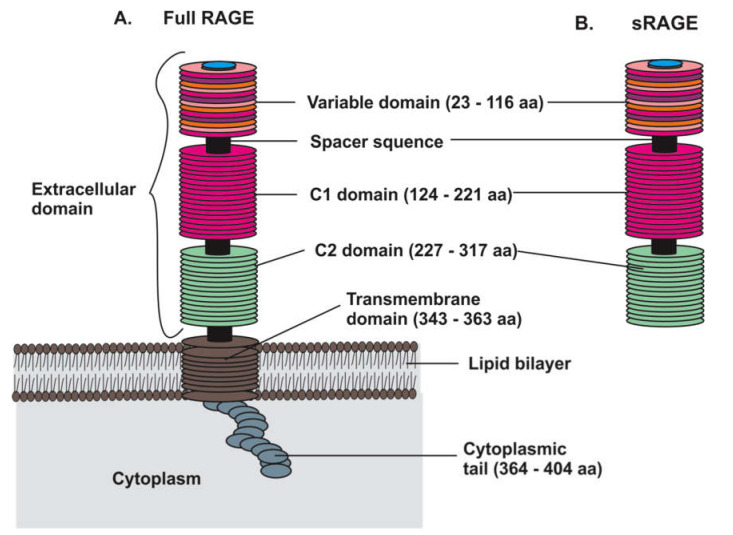
Schematic representation of membrane-bound full-length receptor for advance glycation end products (RAGE) and soluble RAGE (sRAGE). (**A**) Full-length RAGE consists of three extracellular domains (variable domain (V), constant domain 1 (C1), and constant domain 2 (C2)), a single transmembrane domain, and a short cytoplasmic tail. Short spacer sequences are present between the different domains. (**B**) sRAGE is formed by the extracellular domains only and lacks the transmembrane domain and the cytoplasmic tail.

Human RAGE is a multi-domain protein (Figure 1), containing three extracellular domains, a single transmembrane (TM) region, and a short cytoplasmic tail. The variable domain (V), constant domain 1 (C1), and constant domain 2 (C2) constitute the three extracellular domains of RAGE, and they have overall structural folds of variable and constant Ig domains. Full-length RAGE has 404 amino acids and contains a 22 amino acid-long signal peptide for targeting to the cell surface. The length of the different domains is as follows: the V domain comprises amino acids (AAs) 23–116, the C1 domain consists of AAs 124–221, the C2 domain of AAs 227–317, the TM domain of AAs 343–362, and the intracellular domain consists of AAs 363–404 [91,92,93,94,95].

The first successful cloning of RAGE was carried out in 1992 from bovine lung [93]. In this study, HEK293 cells transfected with full-length RAGE cDNA showed a main immunoreactive band on a Western blot at 50 kDa and several other bands between 30 and 40 kDa, suggesting the presence of post-translational modifications of RAGE [93]. Following this study, other groups confirmed the existence of a soluble isoform of RAGE called soluble (s) RAGE (sRAGE). The sRAGE isoform (Figure 1) lacks the transmembrane region and the C-terminal intracellular region [88]. This soluble isoform can result from cleavage of membrane-bound RAGE by proteolytic enzymes or sheddases such as A Disintegrin and Metalloproteinase 10 (ADAM10) [96,97]. sRAGE can also be a consequence of alternative splicing of pre-mRNA [98]. Although other isoforms of RAGE have been described, full-length RAGE and sRAGE are the most frequently observed isoforms [99,100,101,102,103].

**Figure 2:**
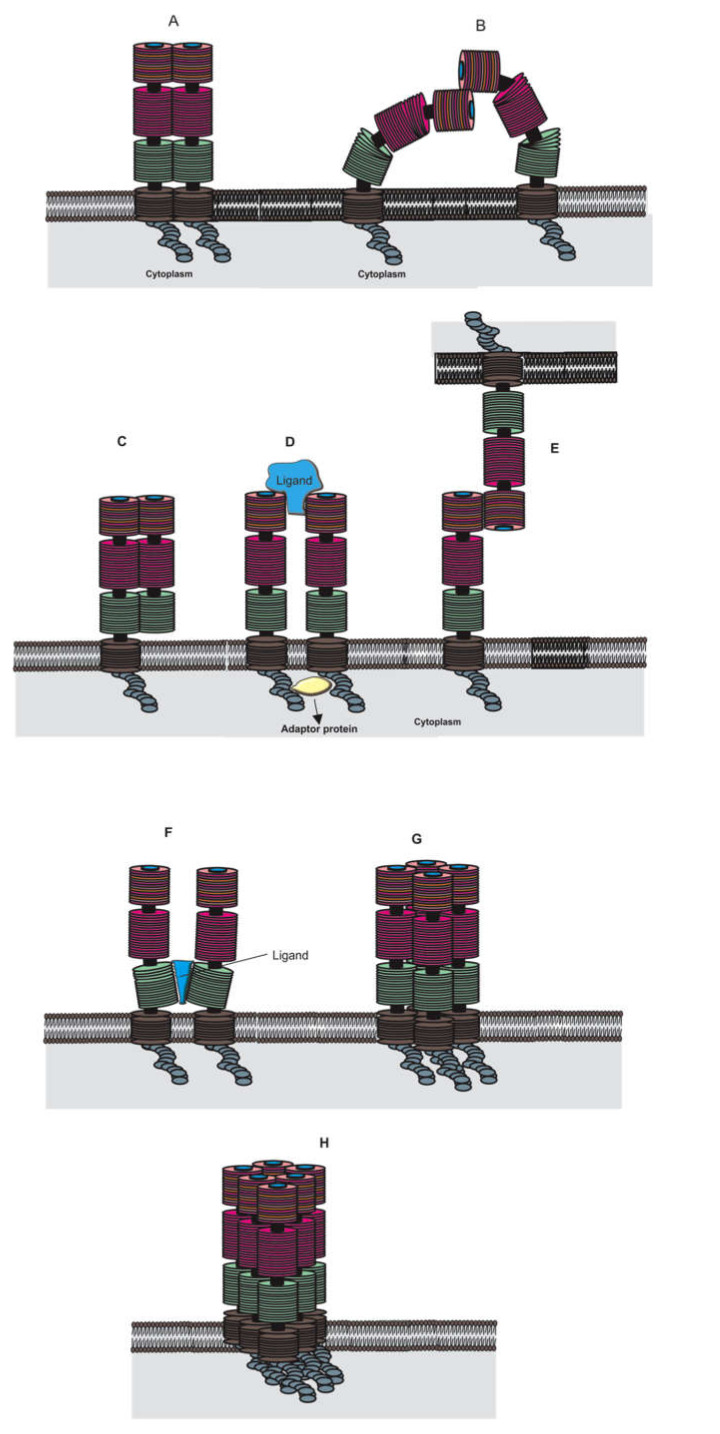
Possible dimeric and oligomeric forms of RAGE. (**A**) RAGE dimers have been suggested to be required for signal transduction [104]; in this representation, all three extracellular domains and the transmembrane domain are involved in dimer formation. (**B**) Dimerization via the V domain only [112]. (**C**) Models for the inhibitory RAGE/sRAGE heterodimer [113]. (**D**) Ligand-induced RAGE homodimers [105]. (**E**) Dimerization via the V domain of RAGE between two different cells [114]. (**F**) Dimerization through a ligand bound to two C2 domains [115]. (**G**,**H**) Other oligomeric forms of RAGE have been proposed as well (tetramers and hexamers) [105].

RAGE can form oligomers; however, the physiological or pathological functions of these oligomers is currently unknown. There is evidence that the homodimerization of RAGE is critical to RAGE-mediated signal transduction [104]. In a previous study, we showed that tetrameric S100B could induce RAGE dimerization as a mechanism of RAGE activation [105]. However, other studies suggest that RAGE may be constitutively expressed as oligomers. In fact, Zong et al. demonstrated that the constitutive dimerization of RAGE is essential for ligand recognition [104]. In addition, all three extracellular domains of RAGE present surfaces that enable dimerization [91,94,106,107]. For instance, the V domain contains hydrophobic patches that allow V domain/V domain interaction [108]. In the C1 domain, two β-strands (L^133^TAGVPNKVGTC^144^ and F^186^TLQSEL^192^) can be further stabilized by dimerization [109]. In the C2 domain, a proline-rich region assembles into an external loop that may be critical for the formation of oligomers, as suggested in a hexameric model of RAGE [108]. Apart from oligomerization mediated by these domains, a recent study has correlated the oligomerization of the transmembrane domain to the presence of GxxxG motifs of these domains [110]. In general, the formation of dimers and higher order oligomers, such as tetramers and hexamers, are facilitated by electrostatic and hydrophobic interactions between multiple RAGE domain surfaces [104,108,111]. Several models of dimeric and higher order oligomeric structures of RAGE have been proposed and are presented in Figure 2.

### 2.2. RAGE Ligands

In physiological conditions, RAGE plays a key role in the resolution of inflammation, tissue repair, and bone homeostasis [116,117]. However, the high expression and activity of RAGE have been incriminated in disease conditions, such as chronic inflammation [113,118,119], diabetes [120,121,122], neurodegeneration [123,124,125], cardiovascular diseases [126,127,128], and cancers [129,130,131]. The activity of RAGE is typically mediated by its ligands but can also be caused by receptor up-regulation. RAGE is a pattern recognition receptor that recognizes Damage-Associated Molecular Patterns (DAMPs), thereby culminating to a downstream pro-inflammatory cascade [104]. RAGE ligands include Advanced Glycation End Products (AGE), calgranulins/S100 proteins, β-amyloid peptides, High Mobility Group Box 1 (HMGB1) protein, transthyretin [132], β2 integrin Mac-1 [133], complement proteins C3a and C1q [134,135,136,137]. The interaction of RAGE with one or more family of these ligands has been implicated in melanoma and other cancers.

### 2.3. S100 Proteins Family

S100 proteins are small EF-hand calcium binding proteins with diverse intra- and extracellular functions (for reviews, see [138,139]). Upon calcium binding, S100 proteins change the conformation and interact with their target proteins that regulate important cellular functions such as cell cycle, cell growth, and migration [138,139]. The role of S100 proteins in cancer is complex, as S100 proteins can have tumor promoter or suppressor effects, depending on the S100 protein and the type of cancer (reviewed in [140,141]). Many members of the S100 protein family are ligands of RAGE [142]. S100 proteins are expressed in many cell types, including melanoma cells. The ability of melanoma cells to secrete S100 proteins was first reported in 1980 [143]. A recent analysis of S100 gene transcripts and clinicopathological data of melanoma patients revealed different expression patterns among different S100 genes, in primary or metastatic melanoma tumors [144]. A first group of genes, including S100A1, S100A13, and S100B was found expressed at high levels in both primary and metastatic melanoma tumors. A second group of S100 genes (S100A2, S100A7, S100A8, S100A9, S100A10, S100A11, and S100P) was highly expressed in primary tumors, but it was expressed at lower levels in metastatic tumors than in control skin [144]. All genes from the second group were strongly correlated with each other, as well as with lymphatic and distant metastases, supporting the role of S100 proteins in melanoma development and suggesting that S100 gene transcript levels could be useful as diagnostic markers [144]. The next sections will provide additional information on specific members of the S100 protein family involved in melanoma (Summarized in Table 3).

#### 2.3.1. S100B

S100B has been described as the “lineage marker” of malignant melanoma [170]. S100B is the most useful [171] and standard [172] biomarker for the follow-up of melanoma patients. S100B serves as a prognostic factor and predictor of overall survival (OS) in melanoma patients. A recent study showed lower S100B levels from patients with stages I and II (primary melanoma) than stage III (regional melanoma) and stage IV (metastatic melanoma), the levels of S100B being the highest in patients with metastatic melanoma [145]. Apart from its use in clinical staging, S100B protein levels are widely used in the clinical management of melanoma patients to determine therapeutic responses [62]. A recent study showed that S100B levels could be used as a prognostic biomarker in patients treated with immune checkpoints inhibitors [65].

S100B has many different binding targets, including RAGE [60,105,113]. We showed that the binding of S100B to RAGE was calcium dependent [105,113], suggesting a link between calcium and RAGE signaling in cells. In a recent study, we also showed that an overexpression of RAGE in the human WM115 melanoma cell line resulted in increased cell migration and invasion [173]. When injected into mice, we showed that the RAGE overexpressing melanoma tumors expressed higher levels of S100B than WM115 control tumors [146], suggesting a positive correlation between RAGE, S100B, and melanoma malignancy. Our data also demonstrated that the RAGE/S100B axis was involved in melanoma development and growth. In addition to its extracellular function, S100B has important intracellular roles. One of these intracellular functions is the regulation of the tumor suppressor p53 protein. The regulation of p53 activity by S100B is complex, and three mechanisms of regulation have been found. S100B can directly reduce p53 activity by binding to the C-terminal oligomerization domain of p53 [147], thereby preventing p53 oligomerization and activation. In vitro data also showed that S100B can inhibit the phosphorylation and regulation of p53 by protein kinase C [148]. In addition, S100B was shown to reduce the tumor-suppressive activities of p53 by down-regulating the expression of p53 downstream effector genes [149].

#### 2.3.2. S100A1

S100A1 is also highly expressed in melanoma tumors, but it differs from S100B in that it is not actively secreted in the serum. Semiquantitative scoring analysis of S100A1 in paraffin-embedded sections of 18 conjunctival nevi, 16 conjunctival melanomas, and 20 uveal melanomas found that S100A1 was more frequently expressed in conjunctival melanoma (71.4% positive cells) and uveal melanoma (88.5%) than in conjunctival nevi (30.6%) [150]. In a different study, the immunohistochemical analysis of melanoma tissues showed a comparatively higher expression of S100A1 in melanoma than in benign melanocytic tumors [151], suggesting that S100A1 may play a critical role in melanoma progression. At the cellular level, S100A1 has been shown to interact with the transient receptor potential melastatin-1 (TRPM-1) channel [153]. TRPM-1 is an important mediator of calcium influx in cells and has been described as a tumor suppressor in melanoma [174]. S100A1 interaction with TRPM-1 could therefore be an important component in melanoma progression. We showed that S100A1 interacts with RAGE in the presence of calcium [152]. A recent study also suggested that S100A1 competes with S100A4 for binding to the V-domain of RAGE [154], suggesting that S100A1/RAGE interaction might influence cell proliferation in melanoma.

#### 2.3.3. S100A2

Earlier studies suggested that S100A2 plays the role of tumor suppressor in melanoma [156,157]. When using a xenograft mouse model, we showed that the overexpression of RAGE in WM115 human melanoma cells implanted in mice resulted in about 1.5 fold higher expression of S100A2 in tumor tissues, as compared to control tumors [146]. However, an analysis of S100 gene transcripts in melanoma tumor samples from different stages showed that the levels of S100A2 transcripts were lower in metastatic melanoma tumors than primary tumors [144]. We had also observed lower levels of S100A2 transcripts in stage III and IV melanoma samples than in control skin samples [155]. These data suggest a complex role of S100A2 in melanoma progression that needs to be further investigated.

#### 2.3.4. S100A4

In many cancers, S100A4 has been shown to stimulate tumor proliferation and metastasis [175,176]. S100A4 is a ligand of RAGE [141,152] and has been shown to stimulate metastasis in various cancer models, including melanoma, through its interaction with RAGE [158,159,177]. Herwig et al. recently showed that the A375 human metastatic melanoma cell line actively secreted S100A4, which acted as an autocrine and paracrine stimulator of RAGE expression [159]. In the same study, the authors reported that the interaction of S100A4 with RAGE resulted in prometastatic activation of A375 cells, with decreased cellular adhesion to fibronectin, increased cell motility, invasiveness, and tumor growth [159]. In a follow-up study, these authors showed that the S100A4/RAGE signaling altered endothelial cell integrity by decreasing tight junction proteins (occludin) and adherence junction protein (E-cadherin) [178]. The authors further showed that S100A4 or RAGE overexpressing A375 cells transmigrated to a higher extent through endothelial cells than control non-transfected A375 cells [178]. All these in vitro data were supported by studies using a mouse model of metastatic melanoma, where mice injected with S100A4 or RAGE overexpressing A375 cells showed higher tumor incidence and mortality than mice injected with the control non-transfected A375 [178]. Additionally, we previously demonstrated that S100A4 levels were significantly higher in RAGE overexpressing WM115 tumors, which were subcutaneously implanted in mice, than in control tumors generated from non-transfected WM115 cells [173]. The results of these studies strongly suggest that the S100A4/RAGE axis is an important contributor to metastasis in melanoma tumors.

#### 2.3.5. S100A6

The expression of S100A6 was described in cutaneous and extracutaneous lesion including melanocytic nevi and malignant melanoma [179]. S100A6 is overexpressed in Spitz nevi, melanocytic nevi, and melanomas [160]; in fact, tissue analysis of melanoma patients revealed that most melanomas showed positive staining for S100A6 [161]. Interestingly, many studies have suggested a role of S100A6 in metastasis, although the exact metastatic mechanism is not specified. An early study revealed a positive correlation between the overexpression of S100A6 and the metastasis of human melanoma cell lines [180]. In another study, gene expression analysis in 45 metastatic melanoma and 20 benign nevi indicated significantly higher levels of S100A6 in metastatic melanoma than in benign nevi [156]. In our xenograft mouse model of melanoma, S100A6 was also found up-regulated in tumors from RAGE overexpressing WM115 melanoma cells compared to tumors from control WM115 cells [146]. Therefore, S100A6 up-regulation may be an important driver in melanomagenesis. In addition, the expression and staining pattern of S100A6 might be useful in distinguishing different forms of melanoma [181].

#### 2.3.6. S100A8/A9

The heterodimeric S100A8/A9 is a complex of two S100 proteins, S100A8 and S100A9. Although S100A8 and S100A9 homodimers have been described [182], the heterodimeric form of these proteins is more frequently observed [139]. Extracellular S100A8/A9 can bind to RAGE and other receptors, thus contributing significantly to the progression of melanoma. In a mouse model of metastatic melanoma, it was shown that uteroglobulin knock-out mice, which naturally overexpress S100A8/A9 in their lungs, developed more metastases in this organ than their wild-type littermates [162]. This study suggested that S100A8/A9 had the ability to attract melanoma cells to the lungs through the activation of RAGE. In a more recent study, the role of S100A8/A9 as a lung attractant for melanoma metastases was confirmed [163]. In this study, the authors showed that a neutralizing antibody against S100A8/A9 could reduce the formation of melanoma metastases in the lungs of mice [163]. S100A8/A9 has also been proposed to be a prognostic marker for metastasis, as well as a predictor of survival and determinant of therapeutic response in melanoma patients [164]. When the expressions of S100A8/A9 proteins were analyzed in melanocytic nevi, primary melanomas, and metastases, higher expression was found in metastases compared to primary melanoma tumors, suggesting that S100A8/A9 is a tumor microenvironment-associated protein that is key to the process of metastasis in melanoma [164].

Other than RAGE, S100A8/A9 recognizes and binds to an array of receptors described as S100 Soil Sensor Receptors (SSSRs) [183]. SSSRs encompass Extracellular Matrix Metalloproteinase Inducer (EMMPRIN), Activated Leukocyte Cell Adhesion Molecule (ALCAM), Toll-like Receptor 4 (TLR-4), Neuroplastin (NPTN) β, and Melanoma Cells Adhesion Molecule (MCAM) [183,184,185]. S100A8/A9/RAGE, S100A8/A9-ALCAM, and S100A8/A9/MCAM axes mediate malignant melanoma progression through the activity of nuclear factor kappa beta (NF-κB) and production of reactive oxygen species (ROS) [185]. Recently, Chen et al. [186] reported the underlining mechanism of melanoma lung tropic metastasis mediated by the S100A8/A9/MCAM axis. This involves the processional activation of MAPKKK8 (Tumor progression locus 2 (TPL2)), ETS translocation variant 4 (ETV4), and induction of matrix metalloproteinase 25 (MMP-25) [186].

#### 2.3.7. S100A13

An expression analysis of S100 genes in melanoma tissues revealed that S100A13 was found highly expressed in melanoma samples [144], although little is known about the contribution of S100A13 to the progression of melanoma. Massi et al. suggested that S100A13 could serve as an angiogenic and prognostic marker in melanoma [165]. Rapidly dividing cancer cells require a high amount of nutrients and oxygen. In order to meet these demands, angiogenesis provides tumor vascularization. The entire process depends on the expression of specific factors such as the Vascular Endothelial Growth Factor (VEGF), and Fibroblast Growth Factors (FGFs). Previous studies have demonstrated a role of S100A13 in the secretion of the FGF, thereby facilitating angiogenesis [166]. In another study, S100A13 was identified as a key player in the resistance of Cutaneous Malignant Melanoma (CMM) to dacarbazine therapy [167], suggesting multiple roles of S100A13 in melanoma metastasis and drug resistance.

#### 2.3.8. S100P

S100P is another protein of the S100 family. It was designated “P” because it was first purified from placenta [187]. Higher levels of S100P were reported in primary melanoma than in nevi and in metastatic melanoma than in primary tumors [168]. A positive correlation was also reported between the expression levels of RAGE and S100P in melanoma tumors [169]. S100P can be localized inside cells or be secreted in the extracellular space; both intracellular and extracellular S100P have been incriminated in tumor proliferation and metastasis [169]. Although RAGE appears to be the receptor for extracellular S100P, the cytoskeletal protein ezrin was found to interact with S100P in the intracellular compartment [169]. Ezrin plays a critical role in cell–cell and cell–matrix contacts. Accumulating evidences suggest that the binding of S100P to ezrin could initiate cell migration in malignant melanoma [188,189].

### 2.4. HMGB1

High Mobility Group Box-1 (HMGB1) was previously referred to as the “chromatin-associated protein” because of its nuclear localization and its activities of modulator transcription and DNA recombination [190,191,192,193]. It is now well established that HMGB1 has also important extracellular functions [194,195,196,197]. Structurally, HMGB1 encompasses three domains, with two identical DNA-binding regions called box A and box B, and a negatively charged C-terminus tail [195]. The major contribution of extracellular HMGB1 to invasiveness and tumor metastasis occurs via its interaction with RAGE [198,199,200]; however, an intracellular role of HMGB1 in tumor progression has also been reported [201].

In tumors, HMGB1 can be released into the extracellular space during necrosis [202] as well as under hypoxic conditions [203]. Extracellular HMGB1 has been shown to interact with RAGE and other cell surface receptors [196,204]. Activation of the HMGB1/RAGE signaling can lead to cell proliferation [205], inflammatory responses [206,207], cell migration [208], chemotaxis, and cytoskeleton reorganization [209].

In melanoma, Tang et al. reported that disruption of the HMGB1/RAGE axis hampered melanoma tumor growth and reduced the synthesis of inflammatory cytokines [210]. This showed that the interaction of HMGB1 with RAGE was critical to maintain an inflamed tumor microenvironment and to tumor growth [210]. The HMGB1/RAGE axis has also been shown to be critical for melanomagenesis. Zhang et al. reported that repeated UV radiation exposure of human melanocytes resulted in an increased secretion of HMGB1 and resistance to subsequent UV-induced apoptosis [211]. Importantly, silencing RAGE in these melanocytes resulted in a decreased secretion of HMGB1 as well as decreased resistance to apoptosis, strongly suggesting that the HMGB1/RAGE axis contributes to the early stages of melanoma development [211]. In a different study, Wang et al. reported that in melanocytes, UV exposure resulted in an increased expression of PD1L1 through the activation of the HMGB1/RAGE axis, and it resulted in significant reduction of the susceptibility of melanoma cells to CD8+ T-cell-dependent cytotoxicity, further demonstrating the important role of the HMGB1/RAGE axis in melanoma development [212]. A recent study also reported that HMGB1 expression levels were higher in patients who did not respond to the immune checkpoint inhibitor ipilimumab than in responding patients, supporting a role of the HMGB1/RAGE axis in enabling a tumor-promoting microenvironment [213].

### 2.5. Advanced Glycation End Products

Advanced Glycation End Products (AGEs) are substances that are formed as a result of non-enzymatic browning or glycation [214]. AGEs are usually formed when reducing sugars react with the amino moiety of proteins in a multi-step reaction involving the generation of intermediates molecules, such as Schiff bases and Amadori products. The overall reaction is called the Maillard reaction [215,216,217,218].

Several studies have demonstrated a role of the AGE/RAGE axis in melanoma progression. In vitro, RAGE was found expressed at higher levels in melanoma cells than melanocytes [219]. In two different studies, AGEs were shown to increase melanoma cell proliferation and migration, tumor growth, and metastasis, in a RAGE-dependent manner [219,220]. Recently, Nakamura et al. showed that melanoma growth and the formation of liver metastases could be reduced when using RAGE-targeting DNA aptamers [221]. The decrease in melanoma growth was associated with a decrease in expression levels of RAGE [221]. In the same study, the authors showed that the exposure of human G361 melanoma cells to AGEs in vitro resulted in increased ROS generation and cell proliferation, as well as increased expression of cyclin D1 and p27, vascular endothelial growth factor (VEGF), and the monocyte chemoattractant protein 1 (MCP-1) [221]. In a similar study, Ojima et al. showed that DNA aptamers could also prevent tumor growth by inhibiting angiogenesis via the disruption of the AGE/RAGE axis [222]. Overall, it was found that the AGE/RAGE axis was critical to melanoma tumor growth and angiogenesis formation.

### 2.6. RAGE Signaling Pathways

**Figure 3:**
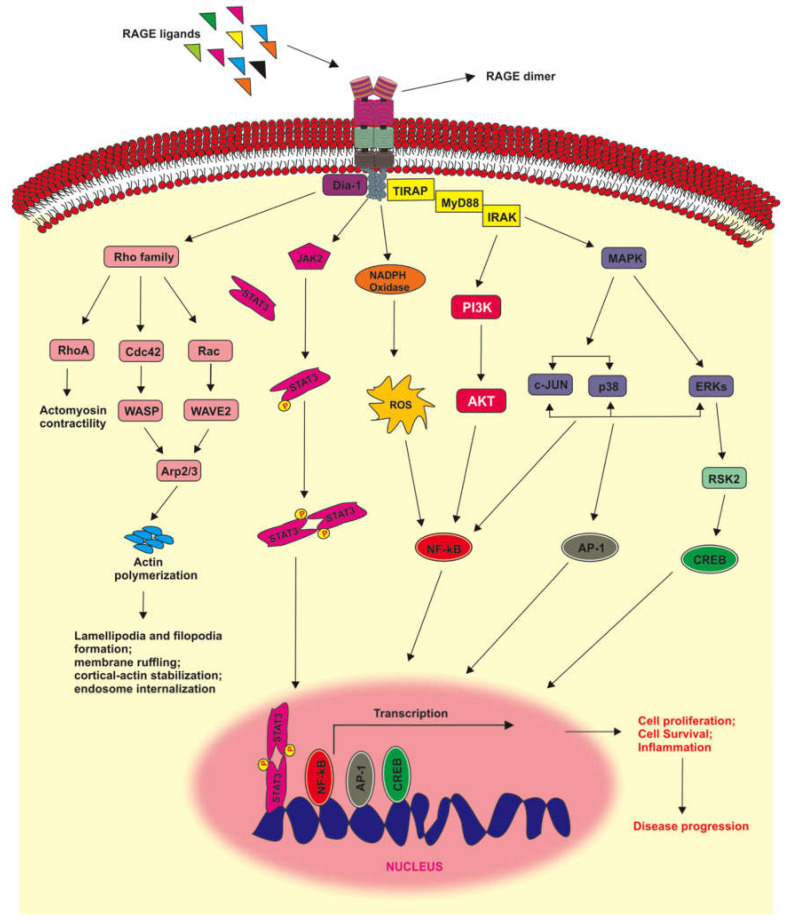
RAGE signaling pathways. The C-terminus cytoplasmic tail of RAGE is crucial to RAGE signaling. Two adaptor proteins interacting with the cytoplasmic tail of RAGE have been identified: Dia-1 and TIRAP [233,234]. RAGE activation leads to increased cell migration through the activation of several members of the Rho family of small GTPases, including RhoA, Cdc42, and Rac-1 [230,233]. The signaling cascades of these proteins (Dia-1/RhoA; Dia-1/Cdc42/Wiskott-Aldrich syndrome protein (WASP)/actin related protein (Arp); Dia-1/Rac/WASP family verprolin-homologous protein-2 (WAVE2)/Arp) lead to actomyosin contractility and actin polymerization [235]. Similarly, RAGE-mediated activation of Janus kinase (JAK) leads to the downstream phosphorylation of Signal transducer and activator of transcription (STAT)3 and its subsequent dimerization resulting in gene transcription [236,237]. Additionally, nicotinamide adenine dinucleotide phosphate (NADPH) oxidase can be activated, leading to ROS generation, which leads to NF-κB activation [238]. RAGE/PI3K/AKT, RAGE/MAPK/c-JUN, RAGE/MAPK/p38, and RAGE/MAPK/Extracellular signal-related kinase (ERK) axes can also result in the activation of NF-κB [239,240,241]. In addition to NF-κB, RAGE has also been shown to signal through AP-1 and cyclic AMP response element-binding protein (CREB) [242,243,244].

RAGE signaling has been incriminated in many diseases including cancer, complications of diabetes, and neurodegenerative disorders [137,223,224,225,226,227]. However, RAGE signaling is also important in many physiological processes such as tissue repair and bone homeostasis [116,228]. Typically, RAGE signaling depends on the type of ligand, its concentration, as well as the cell type, making RAGE a complex receptor to target therapeutically (reviewed in [229]). RAGE signaling is initiated by the interaction of the ligand with the extracellular part of the receptor, mostly the V-domain. Apart from the extracellular domain of RAGE, the short cytoplasmic domain of RAGE is also key in RAGE signaling. Many studies have shown that the deletion of this segment resulted in an inhibition of RAGE signaling, and dominant negative effects were reported as well [230,231,232]. Several adaptor proteins interacting with the cytoplasmic domain of RAGE have been identified. Hudson et al. reported that the FH1 domain of Diaphanous-1 (Dia-1) was essential for the transmission of RAGE signals through the activation of the small GTPases Rac1 and cell division control protein 42 (Cdc42), resulting in cell migration (Figure 3) [233]. Apart from Dia-1, other adaptor proteins transducing RAGE signaling have been identified: Toll-Like Receptor 2/4 adaptors (TIRAP) and myeloid differentiation primary response 88 (MyD88) have also been shown to interact with the RAGE intracellular domain and to mediate RAGE-dependent signal transduction (Figure 3) [234].

Rac-1 and cdc42 proteins belong to the Rho family of small GTPases that are involved in membrane ruffles-initiated cell migration via lamellipodia and filopodia formation [245]. Accumulating evidence suggests that Rac-dependent motility occur via the WASP-family verprolin-homologous protein-2 (WAVE2)/actin-related protein 2/3 (Arp2/3) signaling pathway [235,246]. Another member of the Rho family, RhoA, together with Cdc42, has also been shown to initiate metastasis as a result of the activation of RAGE by S100A4 [247]. The activation of RhoA is critical to cell migration and motility through the phosphorylation of myosin-II light chain and actomyosin contractility (Figure 3) [248,249,250].

One important downstream signaling pathway activated by RAGE is the MAP kinase pathway [239,251]. The MAPK system includes ERK1/2, c-Jun N-terminal Kinases (JNKs, JNK/SAPK), and p38 MAPK (Figure 3) [252]. Activation of this pathway usually results in the activation of NF-κB [251]. Other signaling pathways activated by RAGE have been described: the RAGE/NADPH/ROS [253] and RAGE/PI3K/AKT pathways [254], also leading to the activation of NF-κB (Figure 3) [251]. Most recently, RAGE has also been shown to promote inflammation through the activation of the thioredoxin interacting protein (TXNIP), linking the RAGE/TXNIP axis with the activation of NLR family pyrin domain containing 3 (NLRP3) inflammasone activation (Figure 3) [255,256].

NF-κB is a dimeric transcription factor that belongs to the Rel1 gene family of DNA-binding proteins, and it regulates the transcription of cytokines, growth factors, and anti-apoptotic proteins, thus playing vital roles in proliferation, angiogenesis, metastasis, survival, and immune responses [257]. Another downstream target of RAGE is the Janus kinase 1/2 (JAK1/2)/Signal transducer and activator of transcription (STAT) cascade. Studies have shown that the JAK2–STAT1/STAT3 mediated production of collagen in NRK-49F cells was dependent on the AGE/RAGE axis [236]. In a different study, activation of the AGE-RAGE axis resulted in the upregulation of the immunoproteasome via the JAK2/STAT1 pathway, further confirming the diversity of cellular processes controlled by RAGE and its ligands (Figure 3) [258].

In addition to NF-κB and STATs, other transcription factors are modulated by RAGE activation. Studies have shown that the cyclic AMP response element-binding protein (CREB) can be activated via the RAGE/ERK/RSK2 dependent cascade, resulting in the nuclear translocation of CREB and resultant expression of chromogranin [243]. In another study, inhibition of the HMGB1/RAGE axis suppressed the ERK/p90 ribosomal S6 kinase (p90RSK)/CREB signaling pathway, resulting in the apoptosis in HGC-27 cells [244]. RAGE activation can also lead to the activation of the transcription factor AP-1 though the stimulation of cdc42/Rac-1/JNK [242] or PI3K/Akt/c-Jun [259]. Activation of the RAGE– thioredoxin interacting protein (TXNIP) axis has also been described recently (Figure 3) [255,256].

The influence of RAGE localization on its signaling has been investigated by Popa et al. [260]. In the human primary melanoma cell lines MelJuSo and A375, RAGE was found to have a polarized distribution where RAGE was localized intracellularly and in patches found mostly at membrane ruffles or at other times in cell-to-cell contact sites [260]. However, RAGE localization was more dispersed, with some accumulation at cell profusions, in the metastatic cell lines SK-Me128 and MNT-1 [260]. These authors also identified differences in oligomeric forms of RAGE between the primary and metastatic melanoma cell lines, suggesting that both cellular localization and receptor oligomerization could be important modulators for RAGE signaling [260].

## 3. RAGE Signaling in Melanoma Tumors

An increasing amount of evidence generated from in vitro and in vivo studies suggests that RAGE signaling is an important contributor to the proliferative, inflammatory, and invasive phenotypes of melanoma tumors (reviewed in [261]). Studies have shown that RAGE signaling from both melanoma cells and non-melanoma cells (fibroblasts, immune cells, endothelial cells) present in the tumor microenvironment is an important contributor to melanoma tumor growth.

In tumors, endothelial cells from newly formed blood vessels play major roles by supplying tumor cells with the needed oxygen and nutrients [262]. RAGE is expressed in endothelial cells and in an inflammatory disease model; RAGE activation by its S100B ligand has been shown to increase the expression of the adhesion molecules vascular cell adhesion molecule 1 (VCAM-1) and intercellular adhesion molecule 1 ICAM-1, thereby facilitating the adhesion and recruitment of leukocytes to the site of inflammation [95]. In our earlier study, we observed that RAGE overexpressing WM115 melanoma cells also overexpressed S100B, when compared to control WM115 cells [146]. When secreted into the tumor milieu, S100B produced by melanoma cells could act in a paracrine manner on the nearby endothelial cells, resulting in activation of the S100B/RAGE axis, activation of NF-κB, and the recruitment of immune cells to the tumors, sustaining an inflammatory microenvironment [95]. HMGB1 secreted by melanoma tumors has also been shown to activate endothelial cells through the engagement of RAGE, also resulting in the expression of the adhesion molecules VCAM-1, ICAM-1, and E-selectin, and in the recruitment of immune cells in the tumors, as well as in the secretion of the pro-inflammatory cytokines IL-8 and G-CSF to sustain an inflammatory tumor milieu [263,264].

Macrophages present in the tumor microenvironment also contribute to sustaining inflammation [265]. In macrophages, the engagement of RAGE by its ligands, such as HMGB1, has been shown to activate NADPH oxidase, leading to the generation of reactive oxygen species (ROS) and the downstream activation of NF-κB, the expression of pro-inflammatory cytokines (Il-1, IL-6 and tumor necrosis factor alpha (TNF-α), synthesis of nitric oxide (NO) and superoxide, and resulting in pro-tumoral activities [253,266,267,268]. RAGE signaling in cytotoxic T cells also contribute to fueling inflammation in the tumor microenvironment. Indeed, the HMGB1/RAGE axis was found to influence melanoma tumor growth through the expression of IL-23 and IL-17 from a sub-population of T cells, (gdT cells), resulting in the activation of STAT-3 in a IL-6 dependent manner [210].

In addition to contributing to inflammation in tumors, RAGE signaling promotes immunosuppression in melanoma tumors. A recent study showed that the HMGB1/RAGE axis played a key role in the suppression of cytotoxic T cells activity by increasing the expression levels of PD1L1, leading to PD-1 receptor activation and the down-regulation of cytotoxic T-cells [53,212]. Wild et al. also showed that HMGB1 enhanced the inhibitory functions of Tregs through the activation of RAGE, resulting in an immunosuppressive milieu [269]. Recent studies have established a link between chronic inflammation and immunosuppression in tumors [270], and signaling from the RAGE/ligand axis appears to further support this association. Therefore, the data presented in this review suggest that targeting RAGE in melanoma tumors could have benefits for patients.

## 4. Conclusions

A mounting number of experimental findings and observations has shown that RAGE plays a key role in the progression of melanoma through multiple axes. First, RAGE activation in melanoma cells results in increased cell proliferation and cell migration. As RAGE activation by its ligands results in higher expression of the RAGE receptor itself and of its ligands, RAGE activation can lead to sustained tumor growth. In the tumor stroma, mounting evidence supports the notion that the activation of RAGE expressed on multiple cell types, including endothelial cells, macrophages, and T cells, promotes an inflammatory milieu and sustained inflammation, thereby promoting tumor growth. However, RAGE signaling also controls the immunosuppressive activities of Treg, further facilitating tumor growth and metastasis. Taken together, it appears that targeting RAGE in melanoma tumors with high RAGE expression could be a valid approach to improve current chemo- and immunotherapeutic treatments.

## Figures and Tables

**Table 1:** Different types and groups of melanoma.

Melanoma	Types and Groups		
Cutaneous	Superficial spreading melanoma Nodular melanoma Lentigo malignant melanoma Acral lentiginous melanoma		
Extra-cutaneous	Mucosal	Head and neck Vulvovaginal Anorectal	
	Ocular	Uveal tract	Choroid Iris Ciliary
		Conjunctiva	
	Leptomeningeal	Benign melanocytoma Malignant melanoma	

**Table 2:** Mechanism of action (MOA), overall survival (OS), and date of approval by the Food and Drug Administration (FDA) of selected drugs used for the treatment of metastatic melanoma. The OS data from single drug and recent combination therapies are indicated.

Drug	MOA	OS	Approval Year
Dacarbazine	Alkylating agent	9.1 months [78]	1975
Vemurafenib	BRAF V600E inhibitor	15.9 months [79]	2011
Vemurafenib + Cobimetinib ^1^	BRAF V600E inhibitor MEK inhibitor	22.5 months [80]	2020
Ipilimumab	CTL-4 blocking antibody	19.9 months [81]	2011
Trametinib	MEK inhibitor	14.2 months [82]	2013
Dabrafenib	BRAF V600E inhibitor	13.1 months [82]	2017
Dabrafenib + Trametinib ^1^	BRAF V600E inhibitor MEK inhibitor	25.9 months [83]	2019
Nivolumab	PD-1 antibody	36.9 months [81]	2015
Ipilimumab + Nivolumab ^1^	CTL-4 blocking antibody PD-1 antibody	60 months [81]	2015
Encorafenib + Binimetinib ^1^	BRAF V600E or V600K inhibitor MEK inhibitor	33.6 months [84]	2018
Pembrolizumab	PD-1 antibody	32.7 months [85]	2019

^1^ For the combination.

**Table 3:** S100 proteins and their roles in melanoma.

S 100 Protein	Roles and Main Target Proteins in Melanoma	References
S 100B	Higher expression in metastatic than primary melanoma tumors	[145]
	Used as prognostic marker and indicator of therapeutic responses	[62,65]
	Extracellular S100B activates RAGE	[60,105,113,146]
	Intracellular S100B prevents p53 activation	[147,148,149]
S 100A1	Higher expression in melanoma tumors than benign nevi	[150,151]
	Could modulate melanoma tumor growth through its interaction with RAGE and TRPM-1	[152,153,154]
S 100A2	Lower levels in metastatic than primary tumors A tumor suppressor role has been suggested, but the overall role is complex	[144,155,156,157]
S 100A4	Stimulates melanoma metastasis through RAGE activation Alters endothelial cell integrity	[158,159]
S 100A6	Higher expression in metastatic tumors than in benign nevi Up-regulation in RAGE overexpressing tumors Interacts with RAGE	[142,146,156,160,161]
S 100A8/A9	Higher levels in metastatic than in primary tumors Potential prognostic marker and predictor of survival Promotes lung metastases through the interaction with RAGE and S100 Soil Sensor Receptors (SSSRs)	[162,163,164]
S 100A13	Possible role as angiogenic and prognostic marker Facilitates secretion of angiogenic marker FGF Participates to dacarbazine resistance	[144,165,166,167]
S 100P	Higher levels in metastatic melanoma than in primary tumors and nevi Could promote melanoma metastasis through the interaction with ezrin (intracellular S100P) and RAGE (extracellular S100P)	[168,169]

## Abbreviations

RAGE	Receptor for advanced glycation end product
HMGB1	High Mobility Group Box 1
MSH	Melanocyte stimulating hormone
MC1-R	Melanocortin 1 receptor
CDKN2A	Cyclin-dependent kinase inhibitor 2A
MAPK	Mitogen-activated protein kinase
NRAS	Neuroblastoma RAS viral oncogene homolog
BRAF	V-raf murine sarcoma viral oncogene homolog B1
PI3K	Phosphatidylinositol-3 kinase
mTOR	Mammalian target of rapamycin
NF1	Neurofibromatosis type1
ARID2	AT-rich interactive domain-containing protein 2
TP53	Tumor protein p53
PPP6C	Serine/threonine-protein phosphatase 6 catalytic subunit
DDX3X	DEAD-box helicase 3 X-linked
PTEN	Phosphatase and tension homology
RAC1	ras-related C3 botulinum toxin substrate 1
FBXW7	F-box/WD repeat-containing protein 7
SNX31	sorting nexin 31
PREX2	phosphatidylinositol-3,4,5- trisphosphate-dependent rac exchange factor 2
TACC1	transforming acidic coiled-coil-containing protein 1
KIT	KIT tyrosine-protein kinase
IDH1	isocitrate dehydrogenase1
RB1	retinoblastoma protein 1
SF3B1	splicing factor 3b subunit 1
CTNNB1	catenin (cadherin-associate protein) beta 1
PIK3CA	phosphatidylinositol-4,5-bisphosphate 3-kinase catalytic subunit alpha
CDK4	cyclin-dependent kinase 4
RASA2	ras p21 protein activator 2
WT1	Wilms’ tumor suppressor gene 1
EZH2	enhancer of zeste homolog 2
STK19	serine/threonine-protein kinase 19
SSM	superficial spreading melanoma
AJCC	American joint commission in cancer
OS	Overall survival
DSS	Disease-specific survival
LDH	Lactate dehydrogenase
PD1L1	Programmed cell death receptor 1 ligand 1
PD-1	Programmed cell death receptor 1
IL-2	Interleukin 2
IFN-α	Interferon alpha
FDA	Food and drug administration
CTLA-4	Cytotoxic T lymphocyte antigen 4
MHC	Major histocompatibility complex
TM	Transmembrane
V	Variable
C1	Constant domain 1
C2	Constant domain 2
DAMPS	Damage-associated molecular patterns
AGE	Advanced glycation end products
TRPM-1	Transient receptor potential melastatin-1
SSSRs	S100 soil sensor receptor
EMMPRIN	Extracellular matrix metalloproteinase inducer
ALCAM	Activated leukocyte cell adhesion molecule
TLR-4	Toll-like receptor 4
NPTNβ	Neuroplastin β
MCAM	Melanoma cells adhesion molecule
NF-κB	Nuclear factor kappa beta
ROS	Reactive oxygen species
TPL2	Tumor progression locus 2
ETV4	ETS translocation variant 4
MMP-25	Matrix metalloproteinase 25
VEGF	Vascular endothelial growth factor
FGF	Fibroblast growth factor
CMM	Cutaneous malignant melanoma
MCP-1	Monocyte chemoattractant protein 1
Dia-1	Diaphanous-1
cdc 42	Cell division control protein 42
TIRAP	Toll-like receptor 2/4 adaptors
MyD88	Myeloid differentiation primary response 88
ERK	Extracellular signal-related kinase
JNK	C-Jun N-terminal kinase
TXNIP	Thioredoxin interacting protein
NLRP3	NLR family pyrin domain containing 3
JAK	Janus kinase
STAT	Signal transducer and activator of transcription
CREB	Cyclin AMP response element-binding protein
RSK2	Ribosomal S6 kinase 2
VCAM-1	Vascular cell adhesion molecule 1
ICAM-1	Intercellular adhesion molecule 1
TNF-α	Tumor necrosis factor alpha
NO	Nitric oxide
Tregs	Regulatory T cells
